# Triboelectric Separation for Protein Enrichment of Wheat Flour Compared with Gluten–Starch Mixtures as a *Benchmark*

**DOI:** 10.3390/foods13244075

**Published:** 2024-12-17

**Authors:** Mine Ozcelik, Petra Foerst

**Affiliations:** 1 Food Process Engineering, TUM School of Life Sciences, Technical University of Munich, 85354 Freising, Germany; petra.foerst@tum.de; 2 Process Systems Engineering, TUM School of Life Sciences, Technical University of Munich, 85354 Freising, Germany

**Keywords:** plant protein, protein shift, agglomeration, jet milling, particle tribocharging, electrostatic separation

## Abstract

Triboelectric separation, a solvent-free method, was investigated as a tool for protein enrichment in wheat flour. Gluten–starch model mixtures, flour, and reground flour fractions were evaluated for their separation characteristics (selectivity and efficiency). Mass yield, protein content, particle size distribution, and SEM analysis were used to assess performance. Selectivity and efficiency increased with gluten concentration, peaking at 63% for the 50% gluten mixture, but declined at higher concentrations. The 15% gluten *benchmark* demonstrated effective protein separation, with protein enrichment occurring in the ground electrode fraction and a corresponding depletion in the positive electrode fraction. In contrast, flour and reground flour fractions exhibited reduced separation efficiency, showing protein depletion in both electrode fractions due to agglomeration. The *benchmark* achieved the highest separation efficiency (47%), followed by reground flour (41%) and flour (7%). Finer particles in reground flour enhanced chargeability and GE deposition, while larger agglomerates in flour reduced efficiency, leading to material accumulation in the cups. Pre-milling helped detach protein and starch to some extent but also triggered re-agglomeration. Larger particles were influenced more by gravitational forces. These findings highlight the complexity of wheat flour fractionation and the need to optimize particle size and charge distribution to improve protein enrichment through triboelectric separation.

## 1. Introduction

The rapid growth of the global population has led to an increasing demand for sustainable food sources, making efficient food production methods a top priority. Wheat (*Triticum aestivum*), the second-largest crop globally after corn, reached a production volume of 789 million tons in 2023 [1]. Given its wide application in food products, from bread and cakes to meat analogues, the need for energy-efficient and environmentally friendly methods of protein separation and enrichment has become essential.

The protein content of wheat can fluctuate significantly due to environmental factors such as climate, farming practices, and the use of fertilizers, which directly affect its industrial applications, particularly in dough and baking processes [2]. This variability creates a need for technologies that can adjust the protein content in wheat flour, making dry fractionation techniques essential for optimizing these applications. Wheat kernels consist mainly of starchy endosperm (80–85%), outer layers rich in fiber (12–17%), and wheat germ (3%) [3]. The endosperm contains approximately 85% carbohydrates, mostly starch (80%), which is embedded within a protein matrix predominantly composed of gluten proteins. These gluten proteins are responsible for the elasticity and structural integrity of dough [4,5,6]. This study utilized a model mixture containing 85% starch and 15% gluten, simulating the typical composition of wheat kernels, as a *benchmark* for evaluating protein and starch separation.

Conventional roller milling focuses on reducing particle size through mechanical grinding but does not aim to separate or standardize the key components like protein and starch [7,8]. Roller milling does not achieve a real separation, because it does not utilize differences in the physical or electrostatic properties of these components, leaving protein and starch embedded or mixed due to the structural complexity of wheat flour. However, as the use of fertilizer restrictions increases more and more, resulting in reduced protein levels in wheat, protein standardization has become more essential for industry. This limitation underscores the need for alternative separation methods, such as triboelectric separation, to achieve effective protein standardization. The significance of this work lies in its potential applicability to the flour industry by addressing the critical demand for stabilizing protein content in wheat flour. This research aims to address this gap, offering an innovative solution to selectively enrich protein fractions. By enabling better control over protein levels, this approach can mitigate the challenges posed by environmental variability and regulatory restrictions on fertilizers, ensuring consistent quality in flour-based products. Dry fractionation provides a solvent-free alternative for producing enriched protein and starch fractions, enhancing both process efficiency and quality control while reducing waste. Among dry fractionation techniques, air classification and electrostatic separation have shown considerable potential for plant-based flours. However, air classification, which relies on particle size and density differences, struggles to achieve protein enrichment when particle characteristics are too similar [9,10,11,12,13].

Electrostatic separation techniques, such as corona, induction, and triboelectric charging, charge particles before separating them in an electric field [14]. Triboelectric charging, which remains effective even with small differences in particle conductivity, holds particular promise for protein–starch separation in wheat [15,16]. This process involves electron transfer when two materials come into contact, resulting in one material becoming positively charged and the other negatively charged [17,18]. In triboelectric separation, protein particles tend to acquire a higher charge than carbohydrates due to the presence of ionizable structures such as N- and C-terminus groups and amino acid residues, which allow proteins to charge more readily. In contrast, carbohydrates lack these ionizable groups, making them less prone to charge accumulation [19]. The charging efficiency is influenced by the protein source, as well as factors such as chemical composition, surface chemistry, particle size, shape, surface roughness, moisture content, and the electrical properties of surrounding surfaces, all of which play critical roles in the triboelectric separation process [12,20,21]. The behavior of particles in an electric field depends on their net surface charge, which is influenced by the protein’s isoelectric point—the pH at which the protein carries no net charge [22]. In triboelectric systems, charge transfer primarily occurs through particle–surface interactions (e.g., collisions with the walls of the charging medium) and particle–particle interactions (e.g., contact and friction between particles). These interactions are further enhanced as particles travel through the tribocharging medium, which may include systems such as fluidized beds, vibrating beds, or pneumatically conveyed streams [23,24].

Triboelectric separation has emerged as a promising dry fractionation technique for enhancing protein and fiber content in various crops. Early research by Wang et al. [17] on wheat gluten and polystyrene particles found that smaller particles gained a higher charge due to their increased surface-to-volume ratio, although high water activity negatively affected the charge efficiency by promoting particle agglomeration. Wang et al. [10] later identified lower-than-expected protein enrichment in wheat gluten and starch, likely due to particle agglomeration. Further studies by Sibakov et al. [25] demonstrated the successful enrichment of β-glucane from oat bran using triboelectric separation, while Barakat et al. [26] achieved significant protein enrichment in sunflower oil cakes. Triboelectric separation has been applied across various materials, with protein enrichment reaching 65 g/100 g in lupine and rice bran and up to 20.4% in soy, pea, and chickpea flours through multistage processes [19,27,28,29,30,31]. In our research group, Landauer and Foerst [24,32] reported a 58% protein separation efficiency in starch and whey protein mixtures, emphasizing that higher protein content in the starting material enhances separation efficiency at both electrodes. Recent studies emphasize the critical role of particle size in determining separation efficiency. Smaller particles acquire charge more readily, requiring fewer collisions to reach the same charge-to-mass ratio as larger particles, thereby improving separation efficiency [33,34]. Although triboelectric separation has proven effective across a range of agricultural materials, relatively few studies have specifically focused on gluten proteins. Notably, there is a significant gap in comprehensive research investigating its application to wheat flour. To the best of our knowledge, no systematic evaluation of protein separation in wheat flour or milled flour using triboelectric separation has been conducted, and no standardized *benchmarks* for these separations have been established.

In this study, the authors systematically investigated triboelectric separation in varying gluten–starch mixtures and wheat flour. A gluten–starch mixture that mimicked the composition of wheat flour was used to establish a direct comparison between the two materials. The hypotheses for this work were as follows: (1) Triboelectric separation will effectively differentiate protein and starch fractions in wheat flour based on differences in surface charge, leading to the enrichment of protein in certain fractions. (2) Reducing the particle size of wheat flour through milling will enhance triboelectric separation by increasing the surface charge accumulation and improving the mass-to-charge ratio, thereby facilitating the detachment of protein and starch from the wheat matrix. (3) Wheat flour will exhibit similar separation characteristics to those of a controlled gluten–starch model mixture (85% starch, 15% gluten), serving as a *benchmark* for evaluating triboelectric separation efficiency.

The aim of this study was to investigate the triboelectric separation characteristics of wheat flour and gluten–starch model mixtures, with a particular focus on two aspects: (1) the effect of particle size distribution and the liberation of starch granules from the protein matrix during regrinding on separation efficiency and (2) the distribution of particles on the electrodes, emphasizing how surface charge influences the accumulation of protein and starch fractions across different electrode regions. To achieve this, the factors affecting separation characteristics were assessed by evaluating the mass yield, protein content, and particle size distribution and by using scanning electron microscopy (SEM) to analyze the morphology of the triboelectric separation fractions.

## 2. Materials and Methods

### 2.1. Materials and Preparation of Gluten–Starch Model Mixtures

Vital wheat gluten (G) and starch (S) were used alongside Type 550 German commercial wheat flour to investigate triboelectric separation for protein enrichment in wheat flours. The gluten (typically containing 80–85% protein on a dry basis) and starch were sourced from Kröner Stärke GmbH (Ibbenbüren, Germany), and the wheat flour was obtained from Altdorfer Mühle GmbH (Altdorf, Germany). Gluten–starch model mixtures were prepared by combining gluten and starch at various weight ratios using a food mixer (KitchenAid Artisan 5KSM150, St. Joseph, MI, USA). The mixtures were designed with gluten concentrations ranging from 0% to 100% to evaluate protein and starch separation via triboelectric separation. The selected gluten concentrations were 0%, 15%, 25%, 50%, 75%, and 100%, with 15% gluten chosen as a *benchmark* due to its resemblance to the typical composition of wheat flour (approximately 85% starch and 15% gluten) [3,35].

To evaluate the influence of particle size on separation efficiency, wheat flour was milled using a jet mill (Hosokawa-Alpine, Augsburg, Germany). The jet mill operated at an air pressure of 8 bar, with a feed rate of 5 kg/h and a feeder vibration rate set at 100%. The milled products were collected in filter bags and stored in sealed polyethylene bags at room temperature (20 ± 2 °C). Table 1 lists the sample compositions and their designated names used in this study.

### 2.2. Lab-Scale Triboelectric Batch Separator and Separation Experiment

**Figure 1:**
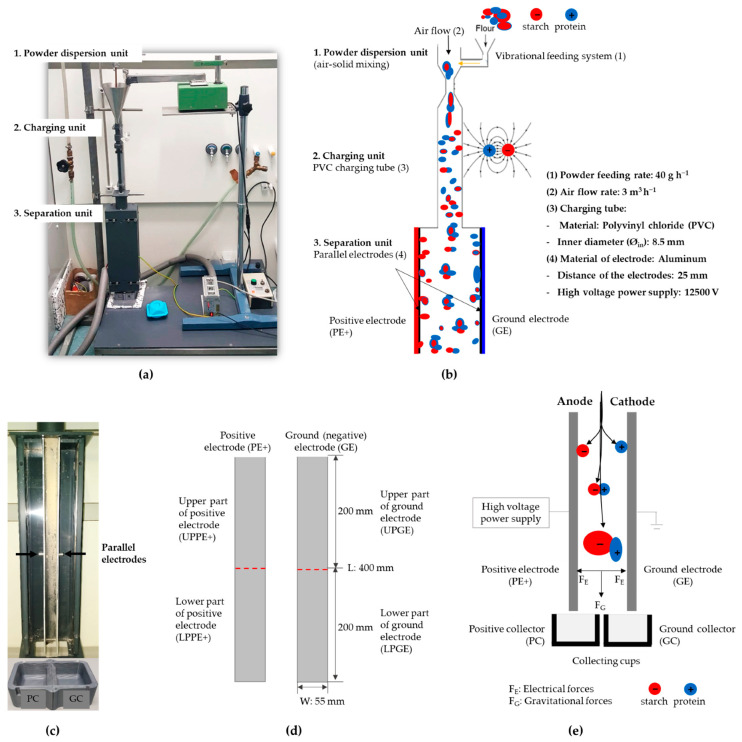
Schematic representation of the custom-built vertical laboratory-scale triboelectric batch separator. (**a**) Photograph of the experimental system. (**b**) Schematic illustration of the separator showing key process parameters. (**c**) Photograph of parallel electrodes within the separation unit. (**d**) Technical drawing of the parallel electrodes. (**e**) Schematic representation of the triboelectric separation mechanism.

A custom-built, vertical lab-scale triboelectric batch separator was used for the separation experiments. The separator was composed of three main sections: (1) the powder dispersion unit, (2) the charging unit, and (3) the separation unit (see Figure 1). Samples were fed into the charging unit using a vibrational feeding system, with a powder feed rate of 40 g/h. Air was introduced from the top of the charging tube at a constant flow rate of 3 m^3^/h, generating turbulent flow (Re~8268) to ensure efficient powder dispersion in the charging unit.

The particles were transported through a straight polyvinyl chloride (PVC) charging tube (230 mm in length, 8.5 mm inner diameter), where triboelectric charging occurred through particle–particle and particle–wall collisions. Upon exiting the charging tube, the particles entered the separation unit, where an electric field was applied between two parallel, oppositely charged plates (400 mm × 55 mm). The electric field was generated using a DC power supply (FuG Elektronik GmbH, Schechen, Germany) set at +12.5 kV (V_0_), leading to an electrical field strength of 500 kV/m.

The charged particles were attracted to the electrode with the opposite polarity based on their charge-to-mass ratio, resulting in the separation of components such as starch and protein. The distance between the parallel electrodes was set to 25 mm, with air flowing in a laminar or transitional regime (Re~2816). One electrode was positively charged (PE+), while the other was grounded (GE). To further analyze the separation behavior, each electrode was divided into upper and lower sections: the upper part of the positive electrode (UPPE+), the lower part of the positive electrode (LPPE+), the upper part of the ground electrode (UPGE), and the lower part of the ground electrode (LPGE). Samples were collected from each section for further analysis.

At the base of the separator, two collection cups were positioned under each electrode: the positive collector (PC) and the ground collector (GC). These cups collected the separated fractions to ensure mass balance throughout the experiment. Particles with a high size and/or low charge, resulting in a low charge-to-mass ratio, were unable to deposit on the electrode surface and instead fell into the cups according to the direction of their existing charge. Before each experiment, the system was cleaned and flushed with dry air. A 40 g sample was used for each run, and after separation, samples were collected from both the electrodes and collection cups. The starting material and the four separated fractions (PE+, GE, PC, and GC) were then analyzed.

Separation behavior was characterized by determining the mass yield, protein content of the fractions, and protein separation efficiency and selectivity. To assess the separation performance, the mass yield and protein content were calculated using Equations (1) and (2):

(1) $$Mass\;yield\;\left(\alpha_{i}\right)(\%)=\frac{m_{i}}{m_{0}}\times 100$$

where $m_{i}$ is the mass of the separation fraction, and $m_{0}$ is the total mass of the starting material before separation.

(2) $$Protein\;content\;\left(\beta_{s,i}\right)(\%)=\frac{m_{p,i}}{m_{i}}\times 100$$

where $m_{p,i}$ is the protein mass of the separation fraction, and $m_{i}$ is the mass of the separation fraction.

Protein separation efficiency and selectivity (relative protein shift) were calculated using Equations (3) and (4):

(3) $$Separation\;efficiency\;(\beta_{e,i})=\frac{m_{p,i}}{m_{p,0}}$$

where $m_{p,i}$ is the protein mass of the separation fraction, and $m_{p,0}$ is the total protein mass of the starting material before separation.

(4) $$Separation\;selectivity\;\left(Relative\;protein\;shift\right)\;(∆\beta_{Rel.,i})=\frac{\beta_{s,i}-\beta_{s,0}}{\beta_{s,0}}$$

where $\beta_{s,i}$ is the protein content of the separation fraction, and $\beta_{s,0}$ is the protein content of the starting material before separation.

The mass balance was calculated using Equation (5):

(5) $$Mass\;balance\;m_{0}\;\left(\sum_{i=1}^{n}m_{i}\right)=m_{PE+}+m_{GE}+m_{PC}+m_{GC}+m_{L}$$

where $m_{0}$ is the total dry mass of the starting material, $m_{PE+}$ is the dry mass of collected samples from the positive electrode, $m_{GE\;}$ is the mass of collected samples from the ground electrode, $m_{PC\;}$ is the mass of collected samples from positive collector, $m_{GC}$ is the mass of collected samples from the ground collector, and $m_{L\;}$ is the mass of the lost material. All experiments were conducted at least in triplicate.

### 2.3. Determination of Moisture Content

The moisture content was determined using ICC Standard 110/1, Determination of the Moisture Content of Cereals and Cereal Products (International Association for Cereal Science and Technology, Vienna, Austria, 1960). Samples weighing 5 ± 1 g were placed in metal drying cups and dried in an oven (UN75, Memmert GmbH., Schwabach, Germany) at 130 ± 3 °C for 2 h. After drying, the samples were transferred to a desiccator and cooled to room temperature before re-weighing. The moisture content was calculated using Equation (6):

(6) $$Moisture\;content\;\left(x_{w,i}\right)\left(\%\right)=\frac{m_{w,i}}{m_{i}}\times 100$$

where $m_{w,i}$ is the mass of the lost water, and $m_{i}$ is the initial mass of the sample.

### 2.4. Determination of Protein Content

The protein content was measured using the Dumas combustion method, employing a Vario Max Cube analyzer (Elementar GmbH, Frankfurt, Germany). Approximately 200 mg of each sample was placed in the analyzer’s auto-sampler, where high-temperature combustion converted nitrogen to nitrogen oxides. The nitrogen oxides were reduced to nitrogen gas, and the total nitrogen content was determined by the instrument. The protein content was calculated using a nitrogen-to-protein conversion factor of N × 5.7. All measurements were performed in duplicate to ensure accuracy.

### 2.5. Determination of Particle Size and Particle Size Distribution (PSD)

The particle size distribution of the samples was measured using a Helos laser diffraction system with a Rodos particle disperser (Sympatec, Clausthal-Zellerfeld, Germany). The distribution was computed by the software, and the median particle size (D_50_) was determined based on the volume distribution.

### 2.6. Scanning Electron Microscopy (SEM)

The morphology of the samples was analyzed using a scanning electron microscope (SEM) (Jeol JSM-7200F, Jeol Ltd., Tokyo, Japan) at an accelerating voltage of 2.00 kV. Samples were coated with a 10 nm gold-palladium layer to enhance conductivity.

### 2.7. Statistical Analysis

All experiments were performed in triplicate. Mean values and standard deviations were calculated for each sample. Statistical analysis was conducted using OriginPro Version 2021 (OriginLab Corporation, Northampton, MA, USA). One-way analysis of variance (ANOVA) followed by Tukey’s multiple comparison test was used to determine significant differences between samples. A *p*-value of less than 0.05 was considered statistically significant.

## 3. Results and Discussion

### 3.1. Triboelectric Separation Characteristics of Gluten–Starch Mixtures and Wheat Flour

#### 3.1.1. Moisture Content and Mass Yield

The mass yield is a key indicator of triboelectric separation efficiency, representing the material collected in each fraction. Yields were calculated on a dry basis to account for moisture content (Table 2) as moisture affects particle charging, especially in hydrophilic materials like gluten. A significant reduction in moisture content (*p* < 0.05), ranging from 2% to 4%, was observed across all fractions compared with the starting material, likely due to the drying effect from airflow within the separator. This airflow exposed particles to friction, promoting evaporation and slightly drying the material. Table 2 shows the moisture content of the tested samples. Although there was a mild drying effect, the average moisture content remained below the 13.8% threshold, above which the tribocharging efficiency decreased, as noted by Wang et al. [17].

**Figure 2:**
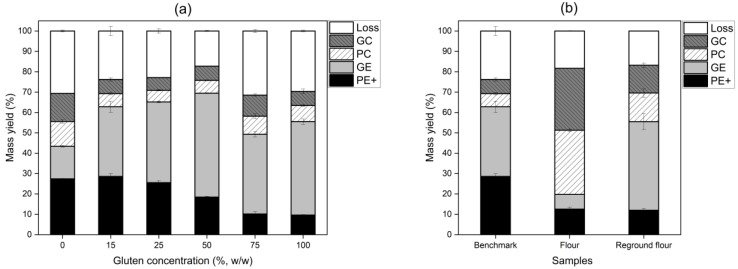
Mass yield of triboelectric separation fractions: positive electrode (PE+), ground electrode (GE), positive collector (PC), and ground collector (GC). (**a**) Gluten–starch mixtures with varying gluten concentrations. (**b**) Flour samples: *benchmark*, flour, and reground flour.

Figure 2a illustrates the mass yield distribution across varying gluten concentrations, while Figure 2b compares the *benchmark*, flour, and reground flour samples.

In all gluten concentrations, the ground electrode (GE) consistently showed a higher mass yield than the positive electrode (PE+), with a peak yield of 51% in the 50% gluten sample. Theoretically, a 50% G model mixture was expected to achieve around 50% mass yield recovery in both the GE and PE+ under optimal conditions. Therefore, obtaining 51% mass from the GE was a strong result, especially considering the high purity of the gluten (~81%), likely due to protein-rich particles acquiring a positive charge and preferentially depositing on the GE [34,35,36]. However, at 0% gluten concentration, where the sample consisted almost entirely of starch, the behavior deviated from this trend. Starch particles, which were negatively charged, preferentially deposited on the PE+ electrode. The absence of protein particles, which exhibited higher charge density, led to a lower mass yield on the GE compared with the PE+. This was consistent with the nature of starch particles, which had lower chargeability due to the lack of ionizable amino groups, resulting in weaker electrostatic separation efficiency.

At higher gluten concentrations (75% and 100% G), the mass yield on the GE declined, contrary to expectations that gluten-dominant mixtures would exhibit higher separation yields. This decrease could be explained by particle–particle interactions during triboelectric charging. As Landauer and Foerst [37] noted, triboelectric charging relies on particles colliding and then separating. In gluten-dominant mixtures, protein particles were more likely to interact with each other rather than with oppositely charged starch particles, leading to fewer opportunities for effective charge transfer. This reduced the overall charge accumulation on gluten particles, thereby diminishing their separation efficiency. Additionally, Coulomb forces played a critical role in the separation process. These forces were proportional to the charge acquired by the particles and were influenced by the particle size, shape, and conductivity. Conversely, in gluten-rich mixtures, the prevalence of protein–protein collisions diminished charge buildup, reducing the effectiveness of Coulomb forces in separating protein from starch. As the contact numbers between particles increased, particularly between protein particles, the charging efficiency diminished, resulting in lower mass yields and protein enrichment on the GE [32,33]. Pelgrom et al. [34] highlighted a similar phenomenon in pea and lupine powders, where higher protein content led to reduced dispersibility in gas flow due to van der Waals interactions, impairing charge transfer and ultimately reducing separation efficiency. The same principle likely applied to the gluten-dominant mixtures in this study as increasing the protein content may have caused reduced dispersibility and decreased separation efficiency. The efficiency and selectivity outcomes related to these phenomena are discussed further in Section 3.2.

These findings contrast with those of Wang et al. [10] who reported lower-than-expected yields in positively charged fractions when the initial protein content was below 65%, with significant material loss. In the current study, the GE mass yield sharply declined at 75% and 100% gluten concentrations, and neither resulted in a corresponding increase in the PE+ yield. Additionally, material loss was higher in these samples, suggesting that gluten-dominant mixtures (above 50% G) were less efficient in separation, likely because they lacked collisions with the other particle type (i.e., starch) to enhance the charging process.

Interestingly, variations in gluten concentration did not significantly (*p* > 0.05) alter the mass yield in the collecting cups, suggesting minimal agglomeration between gluten and starch particles. Most particles were efficiently deposited on the electrodes based on their individual charge properties, without forming large agglomerates that could interfere with separation. The moisture content of the starting material decreased as the gluten concentration increased (Table 2), potentially enhancing protein chargeability at lower moisture levels. Chen et al. [38] similarly observed that reducing the wheat bran moisture from 13% to 7% improved the triboelectric separation efficiency.

For flour samples, the mass yield on the GE was unexpectedly low (7%), with 62% of the total yield collected in the cups. This distribution suggested that flour samples may contain larger starch–protein agglomerates or other particles that were insufficiently charged and, therefore, deposited in the collectors rather than on the GE. This behavior contrasted sharply with the *benchmark* sample, where separation was more efficient. The presence of agglomerates in the flour sample likely hindered separation, causing the particles to remain insufficiently charged or form new agglomerates due to opposite charges.

In contrast, the reground flour showed improved separation performance, with a 32% yield on the GE. The increase suggested that milling reduced particle size, promoting finer particle deposition and liberating more protein from the wheat matrix. This behavior aligned with Xing et al. [29], who reported that milling aided protein detachment, enhancing separation efficiency.

The material loss varied across gluten concentrations, with the highest loss observed in the 0% G (pure starch) sample (45%), followed by 100% G (32%). Interestingly, the material loss at 75% gluten concentration was higher than expected, reaching the largest loss observed at 31.46%. This could be attributed to the particle composition at 75% G, where the mixture of starch and gluten particles led to increased agglomeration and suboptimal charge transfer. At this concentration, the particles may not achieve the optimal charge accumulation necessary for efficient separation, causing more particles to be lost during the process. Other gluten concentrations exhibited losses between 21 and 27%. For both flour and reground flour samples, the material loss was around 20%. Higher losses in pure starch and gluten samples likely resulted from the absence of oppositely charged particles, which, in mixed samples, interacted more effectively with the electric field, leading to better separation. In pure samples, the lack of these interactions likely caused reduced charge accumulation, leading to weaker attraction to the electrodes and more material carried away by airflow.

To better assess these losses, the separator was enclosed in a plastic cover to examine whether material was being dispersed within the system or escaping externally. The results showed that 40–65% of the total loss was dispersed outside the system, while 35–60% remained within the separator. Pure starch, gluten, and reground flour exhibited the highest dispersion outside the system. This relatively high loss was attributed to the airflow in the separator, which likely carried smaller particles, especially given that the system was not airtight. Fine particles, in particular, were more easily carried by airflow and dispersed outside the chamber. These findings were consistent with those of Xing et al. [29], who reported similar material losses (around 25%) during lupine separation.

#### 3.1.2. Particle Size Distribution and Protein Content

**Figure 3:**
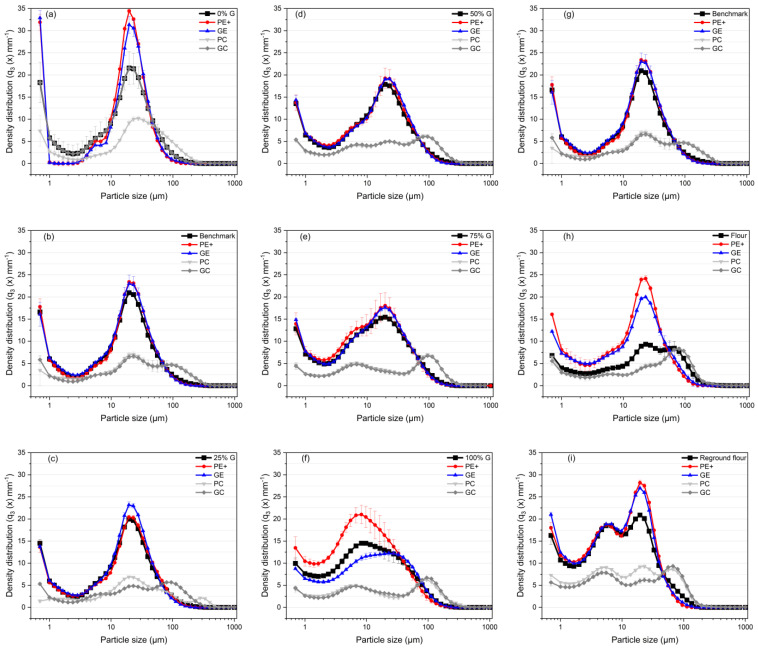
Particle size distribution (PSD) curves of gluten–starch mixtures, flour samples, and their triboelectric separation fractions: PE+, GE, PC, and GC. (**a**) 0% gluten. (**b**) 15% gluten (*benchmark*), (**c**) 25% gluten, (**d**) 50% gluten, (**e**) 75% gluten, (**f**) 100% gluten, (**g**) *benchmark*, (**h**) flour, and (**i**) reground flour.

**Figure 4:**
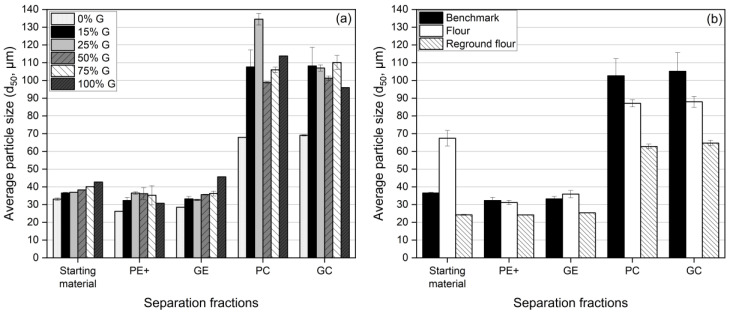
Average particle size ($d_{50}$) of triboelectric separation fractions: PE+, GE, PC, and GC. (**a**) Gluten–starch mixtures with varying gluten concentrations. (**b**) Flour samples: *benchmark*, flour, and reground flour.

Figure 3 and Figure 4 present the particle size distribution (PSD) and d_50_ values for each fraction. Figure 3b,g both present the same sample, 15% G (*benchmark*), to maintain the logical flow of the presented samples. This sample is shown twice for consistency.

**Figure 5:**
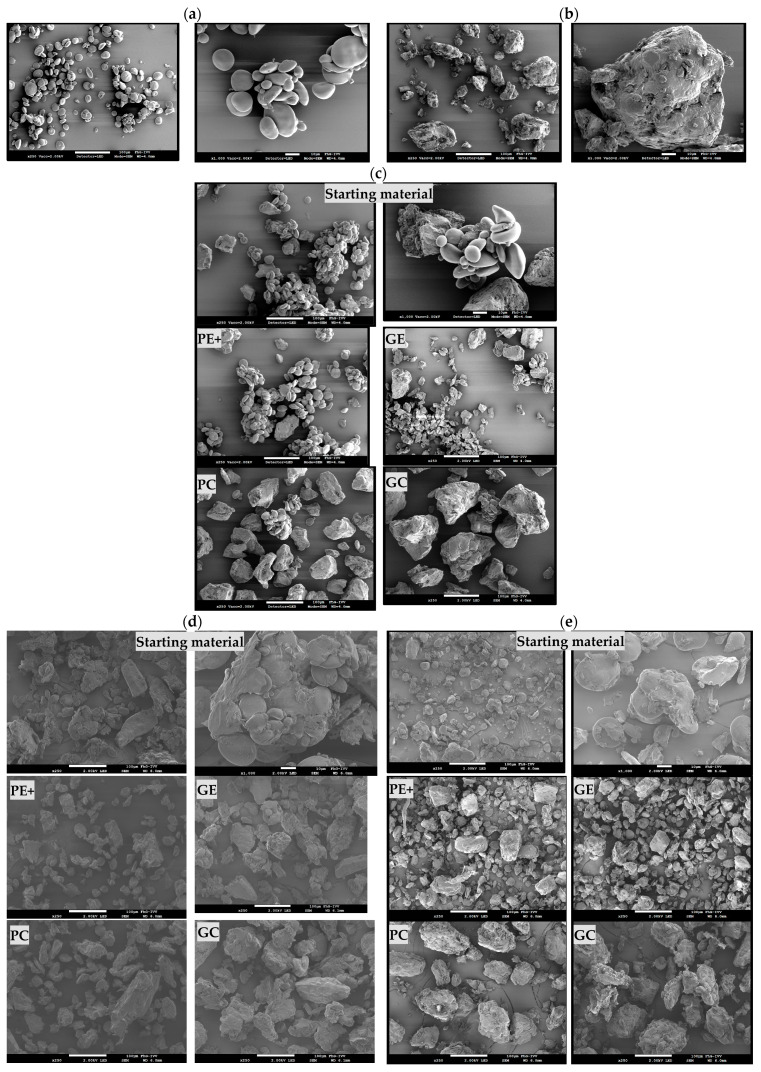
SEM images of the starting material and triboelectric separation fractions collected from PE+, GE, PC, and GC. (**a**) Starch, (**b**) gluten, (**c**) *benchmark* sample and its fractions, (**d**) flour and its fractions, and (**e**) reground flour and its fractions.

The PSD curves revealed that the GE and PE+ fractions generally exhibited narrower peaks, indicating a more uniform particle size distribution. In contrast, the PC and GC fractions displayed broader PSD curves, reflecting a wider range of particle sizes. This suggested that larger particles, with a lower charge-to-mass ratio—possibly due to agglomeration or ineffective charging—were collected in these fractions [26]. As the gluten concentration increased, smaller particles were consistently collected on the electrodes, particularly on the ground electrode (GE). In the 50% gluten sample, for example, the GE fraction had a d_50_ of 35.65 µm, indicating effective separation of finer particles, while the GC fraction showed a d_50_ of 101.28 µm, reflecting that larger particles remained in the collectors. These trends aligned with the protein content results in Table 3, where the GE consistently had higher protein content across all samples, reaching 48.43 g/100 g dry matter in the 50% gluten sample, while the collectors (PC and GC) retained particles with lower protein content. This demonstrated that the finer, protein-rich particles were more likely to be deposited on the electrodes due to their higher charge accumulation. The SEM images (Figure 5, discussed in detail in Section 3.1.3) confirmed this observation, showing that the GE and PE+ fractions exhibited smaller, more uniform particles compared with the broader size distribution seen in the PC and GC fractions.

The particle size distribution and d_50_ data also highlight significant differences between the flour, *benchmark*, and reground flour samples. The flour sample exhibited broader PSD curves, especially in the PC and GC fractions, with d_50_ values of 87.1 µm (PC) and 87.97 µm (GC). The presence of larger particles suggested less effective separation, likely due to agglomeration or inadequate charging. In contrast, the *benchmark* sample exhibited narrower PSD curves, reflecting a more uniform particle size distribution. Although the d_50_ values in the collectors were slightly higher (102.63 µm for PC and 105.16 µm for GC), the uniformity of particle sizes suggested improved charging behavior during separation. Expectedly, the reground flour showed finer particle sizes overall, particularly in the GE fraction, where the d_50_ was 24.19 µm, suggesting that milling improved the separation of smaller, protein-rich particles.

As discussed in Section 3.1.1, it is important to note that the reground flour exhibited a higher mass yield in the GE, even though the protein content of the GE for both the reground flour and the flour sample was similar at 11.05 g/100 g dry matter, showing no significant difference. This indicated that particle size reduction through milling enhanced separation performance by allowing more particles to be charged and deposited on the electrodes, making the separation efficiency more pronounced. These findings aligned with Pelgrom et al. [34] and Wang et al. [26], who emphasized that proper milling was crucial for effective triboelectric separation. Milling not only disassociated cellular components but also reduced particle size, which promoted better charge accumulation. However, it is important to note that if particles become too small, van der Waals interactions, lipid exposure, or reduced collision frequency can lead to re-agglomeration, hindering the charging process [39].

The protein content results across all samples aligned with the expected values based on the gluten concentrations in the model mixtures. In all tested model mixtures, the GE consistently yielded higher protein content than the starting materials, indicating that when starch and gluten were present as separate particles in the mixtures, proteins were more readily charged and deposited on the GE. However, for both flour and reground flour, the protein content in the GE showed no significant difference (*p* > 0.05) from the starting material, remaining at approximately 11%. On the other hand, the protein content in the PE+ fraction decreased to 9.9%, while the collecting cups exhibited a significantly higher protein content of 12%. These results suggested that larger particle agglomerates in the flour sample were not fully separated and were instead collected in the cups. For the reground flour, finely ground particles may have led to re-agglomeration, reducing their charge or forming larger clusters. These findings underscore the critical role of particle size and charge balance in triboelectric separation. Reducing particle size does not necessarily improve efficiency; rather, an optimal particle size must be achieved to enhance separation efficiency.

#### 3.1.3. SEM Images

Figure 5 illustrates the SEM images of key components from the gluten–starch model mixtures, alongside the *benchmark*, flour, and reground flour samples, highlighting their morphological characteristics. Figure 5a,b demonstrate pure starch and pure gluten at two magnifications (×250 and ×1000), along with the starting material of other samples, providing a clear view of their distinct morphological characteristics. Pure starch (Figure 5a) appeared smooth and spherical, with a relatively uniform particle size (d_50_: 33 µm). This smooth, spherical nature likely contributed to lower charge accumulation, which was reflected in the relatively low mass yield of 27% on the PE+ for the 0% gluten sample. In contrast, pure gluten (Figure 5b) exhibited a dense, irregular, and flattened structure, which facilitated better charge accumulation and led to a higher mass yield of 47% on the GE for the 100% gluten sample. This distinct structural difference between starch and gluten explained the better adhesion of gluten particles to the electrodes [33].

The SEM images of the 15% G model mixture (*benchmark*) in Figure 5c show gluten–starch aggregates forming clusters, where smaller starch particles surrounded larger gluten particles. This arrangement differed from the structure observed in wheat flour, where the components were more tightly integrated. This observation aligned with the findings of Xing et al. [40], where oppositely charged particles were attracted to each other, with small starch particles carrying larger gluten particles, likely due to gluten acquiring a negative charge in the starch-dominated environment. This was further supported by the particle size distribution data (Figure 3), which showed that starch particles (d_50_: 33 µm) were smaller than gluten particles (d_50_: 43 µm). On the PE+, starch was the dominant, negatively charged component, while some gluten particles were also present, likely attracted by the starch environment. In contrast, the GE was dominated by gluten particles, with some starch particles remaining embedded in the matrix.

The higher chargeability of protein may influence this starch–gluten interaction, leading to co deposition in specific regions as oppositely charged particles tend to associate due to Coulombic interactions, especially in smaller particles with higher specific charge [41].

For the flour sample (Figure 5d), starch particles were embedded within a tightly packed protein matrix. This differed from the *benchmark* mixture, where protein and starch were more loosely associated. The tighter binding in flour may be due to the presence of lipids, fibers, or pentosans, which enhance cohesion between starch and protein, making separation more challenging [42]. Reground flour showed higher mass deposition on the GE, likely due to smaller particle sizes and better detachment of protein from the clusters, resulting in more effective separation.

Smaller particles in the reground flour sample, due to their higher surface-to-volume ratio, experienced more frequent collisions and greater surface contact, enhancing triboelectric charging. However, even after milling, some protein and starch particles remained partially attached to each other. While reducing the particle size increased the charge-to-mass ratio and enhanced separation, over-milling could lead to the formation of agglomerates, where oppositely charged particles reattached, reducing selectivity. Therefore, a balance was crucial—milling enhanced separation efficiency, but excessive milling could have adverse effects.

In all samples, the collecting cups (PC and GC) contained larger particles (d_50_: 70–135 µm), which were insufficiently charged and predominantly influenced by gravitational forces. Larger gluten particles and agglomerates, due to strong intermolecular forces, were more likely to re-agglomerate and remain in the collecting cups. These findings were consistent with previous studies that highlighted the role of particle size, chargeability, and intermolecular forces in the separation process [43,44]. Even when these aggregates were initially separated during charging, they may re-agglomerate within the laminar flow of the separator, becoming neutral and eventually collected in the bins.

### 3.2. Triboelectric Separation Selectivity and Efficiency of Gluten–Starch Mixtures and Wheat Flour

**Figure 6:**
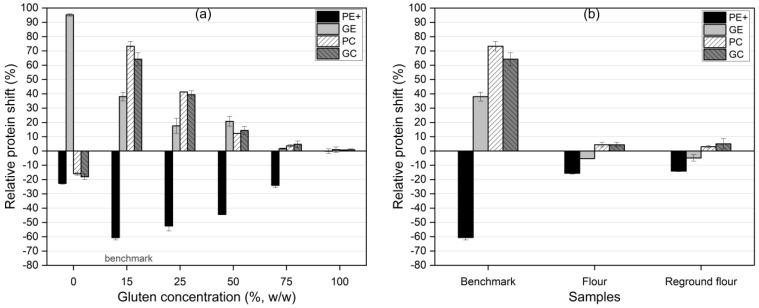
Relative protein shift (separation selectivity) of triboelectric separation fractions (PE+, GE, PC, and GC). (**a**) Gluten–starch mixtures with varying gluten concentrations. (**b**) Flour samples: *benchmark*, flour, and reground flour.

The goal of this study was to achieve efficient protein separation by shifting protein particles from the feed material to the ground electrode (GE). Efficient separation would be demonstrated by a positive shift in the protein content on the GE, accompanied by a corresponding depletion of protein on the PE+. As shown in Figure 6, the relative protein shift varied significantly across gluten concentrations and flour samples.

For gluten–starch mixtures, the 0% gluten (pure starch) sample showed the highest efficiency, with 95% of the protein shifting to the GE and a −23% shift on the PE+. This result suggested that the low protein content in the starch-dominant mixture allowed for a more efficient charge buildup, resulting in optimal separation. A clear trend emerged between 15% and 75% gluten concentrations, where increasing gluten content reduced the efficiency of protein separation from the PE+. The 15% gluten *benchmark* sample showed optimal efficiency, with a significant increase in protein content on the GE (15%) and a strong depletion of protein on the PE+ (−61%), indicating effective protein movement to the GE. However, at higher gluten concentrations (75% and 100%), the separation efficiency declined. This decrease was likely due to the formation of larger protein aggregates, whose net charge was less and more prone to gravitational settling in the PC and GC fractions rather than adhering to the electrodes.

**Figure 7:**
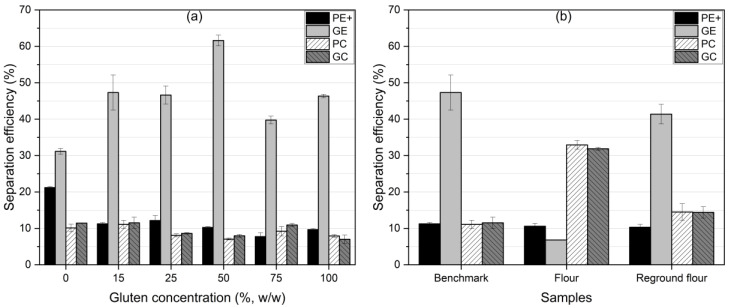
Separation efficiency of triboelectric separation fractions (PE+, GE, PC, and GC). (**a**) Gluten–starch mixtures with varying gluten concentrations. (**b**) Flour samples: *benchmark*, flour, and reground flour.

Figure 7 shows that separation efficiency at the GE was relatively stable between 15 and 25% gluten concentrations, peaking at around 62% for the 50% gluten mixture. This high efficiency was likely due to gluten’s higher chargeability at moderate concentrations. However, beyond 50% gluten, the efficiency decreased, likely due to protein–starch interactions or the formation of agglomerates that impeded charge accumulation and lower separation selectivity. This behavior aligned with findings by Pelgrom et al. [34], who reported that higher protein content reduced dispersibility in the airflow, hindering charge accumulation and decreasing separation efficiency.

In the flour and reground flour samples, separation was more complex due to the presence of non-protein components like fibers and lipids, which interfered with protein chargeability and reduced both selectivity and efficiency [42]. This resulted in a sharp drop in protein capture on the GE for the flour sample, with an efficiency of only 6.8%. Regrinding, however, significantly improved the separation efficiency, increasing it to 41.3%—closely aligning with the *benchmark* value of 41.4%. This improvement could be attributed to better protein liberation from the matrix during milling, as well as finer particle sizes and enhanced chargeability. However, milling may also promote agglomeration, which could reduce selectivity due to the influence of gravitational forces on larger particles.

These findings underscored the importance of particle size and charge balance in triboelectric separation. Finer particles were more likely to acquire charge and adhere to the GE, enhancing both protein enrichment and separation efficiency. However, increasing the gluten concentration beyond 50% decreased efficiency as larger protein particles formed agglomerates that disrupted the charge accumulation and separation process.

### 3.3. Particle Deposition on Electrode Surfaces

As outlined in Section 2.2 and illustrated in Figure 1d, particle deposition on different sections of the electrode surfaces was analyzed to assess variations in particle composition. The electrodes were divided into upper and lower sections for both the positive electrode (PE+) and the ground electrode (GE). It was hypothesized that larger particles, despite having a lower charge-to-mass ratio, would preferentially deposit on the upper sections of the electrodes due to their proximity to the charging tube. In contrast, larger particles collected in the cups had insufficient charge to adhere to the lower sections of the electrodes. As their charge-to-mass-ratio was low, these particles were more likely to fall into the collection cups. Smaller, oppositely charged particles, such as starch and protein, tended to re-agglomerate and deposit more randomly on the lower sections, influenced by their net charge distribution and the airflow pattern in the charging section [45].

**Figure 8:**
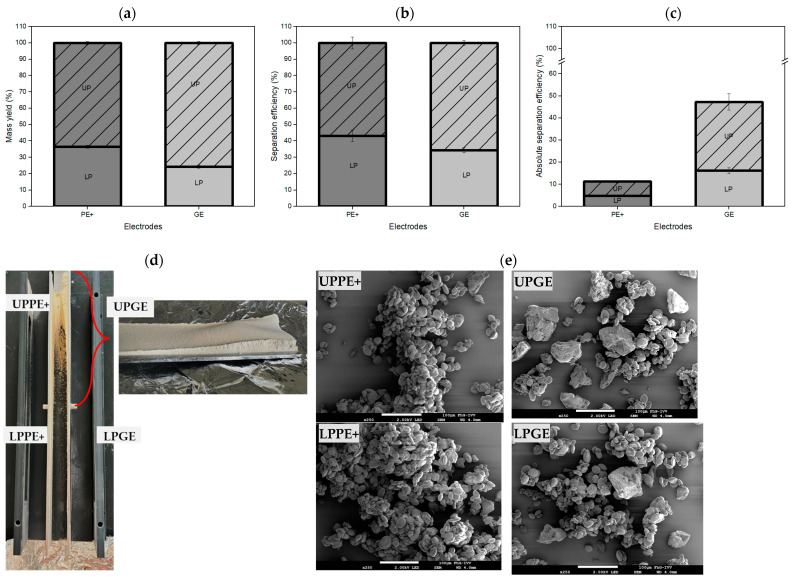
Distribution of mass yield and separation efficiency between upper and lower sections of PE+ and GE for the 15% gluten (*benchmark*) sample. (**a**) Mass yield across electrode sections. (**b**) Separation efficiency across electrode sections. (**c**) Absolute separation efficiency across electrode sections, showing the total efficiency per section relative to the starting material. (**d**) Photograph of electrodes post-separation. (**e**) SEM images of electrode sections.

The mass yield for the upper part of the PE+ was 64% (Figure 8a), while the separation efficiency was 57% (Figure 8b). The absolute separation efficiency further underscored the distinct deposition patterns on the electrode sections. On the PE+, the absolute separation efficiency was higher on the upper section (UPPE+, 6.4%) compared with that on the lower section (LPPE+, 4.8%), indicating that highly charged protein particles adhered preferentially to the upper region due to their superior charge-to-mass ratio (Figure 8c). The SEM images of the upper PE+ section (Figure 8e) showed mostly small starch clusters with a few gluten–starch agglomerates. On the GE, 76% of the total mass was deposited on the upper section, with a separation efficiency of 65%. Similarly, on the GE, the upper section (UPGE) exhibited the highest absolute separation efficiency at 31.1%, significantly exceeding the lower section (LPGE) at 16.2%. These results aligned with the hypothesis that highly charged particles, especially gluten, were more likely to accumulate in the upper sections due to enhanced chargeability and preferential adhesion. A photograph of the electrodes after separation (Figure 8d) revealed a thicker layer of particles on the upper GE section, while the lower section had a much thinner layer. SEM images (Figure 8e) from the GE showed that larger gluten particles were primarily captured on the upper section, while the lower section contained smaller gluten particles and some starch.

This deposition pattern suggested that highly charged particles, particularly gluten with its higher charge-to-mass ratio, tended to adhere more readily to the upper sections of the electrodes. The thick deposition layer on the surface resulted from the combined effects of Coulomb forces, van der Waals forces, and polarization-induced adhesion [46]. Upon contact with the electrode, the particle’s charge dissipated, but polarization induced by the electric field formed a dipole, sustaining adhesion. Van der Waals forces further enhanced retention, especially as more particles settled on the initial layer. These particles, being in contact with non-conductive material, experienced slower charge dissipation [47]. If particles were restricted in movement, such as when part of an agglomerate, their primary charge may persist, further increasing adhesion [39,48].

The thicker deposition observed on the upper GE section could be attributed to several factors. Larger, protein-rich particles, with their higher charge-to-mass ratio, acquired more charge and adhered more strongly to the electrode surface. Increased surface contact during particle collisions amplified Coulomb forces, promoting particle accumulation [41]. The stronger electric field near the upper electrode section also drew these highly charged particles immediately after they passed through the charging tube. Irregular protein particle shapes and flat surfaces increased the contact area, reinforcing retention. As more particles deposited, the initial layer provided a conductive path for subsequent layers, resulting in the pronounced deposition seen on the upper section of the GE (Figure 8b).

## 4. Conclusions

This study highlights the critical role of particle size and the liberation of protein from starch in triboelectric separation for protein enrichment in wheat flour. Reground flour, with smaller particles, exhibited improved protein separation efficiency due to better liberation of starch and protein bodies from the matrix, leading to enhanced chargeability and more effective deposition on the ground electrode (GE). However, our findings partially confirmed hypotheses (2) and (3). Hypothesis (2) was supported, as reground flour with smaller particles showed improved protein separation efficiency due to better liberation of starch and protein, which enhanced chargeability and deposition on the ground electrode (GE). Hypothesis (3) was also partially confirmed, as the gluten–starch model mixture showed better separation efficiency than regular flour. However, the presence of additional components such as lipids in the flour hindered the separation. These non-free components interfered with the charge transfer and protein enrichment. The *benchmark* sample achieved a separation efficiency of 47%, while reground flour reached 41%, compared with only 7% for flour. This result highlighted that aggregated particles, which were difficult to disintegrate through electrostatic forces, significantly reduced the protein enrichment in the GE, especially in the flour sample.

Our findings also indicate that protein enrichment in wheat flour is more challenging than in other cereals, such as peas and legumes, due to the complex matrix of wheat. Nonetheless, the use of pre-treatment methods, such as milling, showed promising potential for enhancing protein fractionation in wheat flour.

Notably, the efficiency of the collecting cups for the flour sample reached 33%, suggesting that recycling the material collected in the cups through additional separation cycles could enhance overall efficiency. This emphasized the need to optimize particle size and prevent excessive agglomeration to improve both selectivity and efficiency.

Future research should focus on refining key parameters such as mass feed rate and airflow conditions, as well as exploring alternative charging tube designs to further improve separation efficiency. Techniques like ultrasonic sieving could also be explored to enhance the dispersability of flour prior to triboelectric separation. Additionally, investigating wheat flours with varying particle size distributions could provide valuable insights into optimizing triboelectric separation for different applications. Last, improvements in separator design, particularly enhancing the electric field strength in the middle to lower parts of the electrodes—where thinner layer deposition was observed—could help capture lower-charged or larger particles more effectively. A hyperbolic electric field design may offer a promising solution to achieve this.

## Figures and Tables

**Table 1:** Compositions of gluten–starch mixtures and flour samples with their designated names.

Sample Composition	Designated Name
0% (*w*/*w*) gluten + 100% (*w*/*w*) starch	0% G
15% (*w*/*w*) gluten + 85% (*w*/*w*) starch	15% G; *benchmark*
25% (*w*/*w*) gluten + 75% (*w*/*w*) starch	25% G
50% (*w*/*w*) gluten + 50% (*w*/*w*) starch	50% G
75% (*w*/*w*) gluten + 25% (*w*/*w*) starch	75% G
100% (*w*/*w*) gluten + 0% (*w*/*w*) starch	100% G
Flour (Type 550)	Flour
Jet-milled flour (Type 550)	Reground flour
Sample before separation	Starting material

**Table 2:** Moisture content of the tested gluten–starch mixtures and flour samples.

Sample Name	Moisture Content (%)				
	Starting Material	Positive Electrode (PE+)	Ground Electrode (GE)	Positive Collector (PC)	Ground Collector (GC)
0% G	13.56 ± 0.03 ^a^	9.80 ± 0.88 ^b^	9.78 ± 0.56 ^b^	9.65 ± 0.34 ^b^	9.36 ± 0.69 ^b^
15% G (*benchmark*)	12.47 ± 0.48 ^a^	9.05 ± 0.47 ^b^	8.89 ± 0.15 ^b^	8.80 ± 0.40 ^b^	8.71 ± 0.26 ^b^
25% G	12.52 ± 0.04 ^a^	8.26 ± 0.01 ^b^	8.29 ± 0.20 ^b^	7.63 ± 0.23 ^b^	8.06 ± 0.15 ^b^
50% G	11.62 ± 0.05 ^a^	8.18 ± 0.07 ^b^	8.29 ± 0.11 ^b^	7.73 ± 0.34 ^b^	7.85 ± 0.24 ^b^
75% G	10.48 ± 0.00 ^a^	8.39 ± 0.94 ^b^	7.56 ± 0.28 ^c^	7.27 ± 0.16 ^c^	7.15 ± 0.17 ^c^
100% G	9.79 ± 0.14 ^a^	8.72 ± 0.22 ^b^	8.72 ± 0.36 ^b^	8.43 ± 0.15 ^b^	8.52 ± 0.09 ^b^
Flour	11.80 ± 0.04 ^a^	9.44 ± 0.00 ^b^	9.16 ± 0.03 ^b^	9.70 ± 0.00 ^b^	9.62 ± 0.02 ^b^
Reground flour	11.55 ± 0.17 ^a^	7.98 ± 0.42 ^b^	8.36 ± 0.64 ^b^	8.44 ± 0.54 ^b^	9.72 ± 0.01 ^b^

The average values are reported with their corresponding standard deviation, and n = 3. Statistically significant differences (*p* < 0.05) between the different samples within the same column and within the same row separation fractions are indicated by different superscript letters.

**Table 3:** Protein content of samples and triboelectric separation fractions (positive electrode, ground electrode, positive collector, and ground collector).

Sample	Protein Content (g/100 g Dry Matter)				
	Starting Material	Positive Electrode (PE+)	Ground Electrode (GE)	Positive Collector (PC)	Ground Collector (GC)
0% G + 100% S	0.40 ± 0.03	0.31 ± 0.02	0.79 ± 0.07	0.33 ± 0.01	0.34 ± 0.02
15% G + 85% S (*benchmark*)	9.84 ± 0.20	3.88 ± 0.24	13.57 ± 0.29	17.05 ± 0.62	16.16 ± 0.65
25% G + 75% S	20.38 ± 0.15	9.69 ± 0.80	23.96 ± 0.90	28.81 ± 0.22	28.09 ± 0.08
50% G + 50% S	40.12 ± 0.44	22.29 ± 0.21	48.43 ± 0.85	45.03 ± 0.55	45.63 ± 0.98
75% G + 25% S	60.56 ± 0.04	45.99 ± 1.00	61.56 ± 0.25	62.77 ± 0.52	63.87 ± 0.80
100% G + 0% S	81.24 ± 1.20	81.14 ± 0.06	82.04 ± 0.30	81.74 ± 0.96	82.24 ± 1.12
Flour	11.67 ± 0.04	9.85 ± 0.04	11.25 ± 0.06	12.18 ± 0.15	12.23 ± 0.06
Reground flour	11.61 ± 0.16	9.98 ± 0.09	11.05 ± 0.11	11.97 ± 0.07	12.26 ± 0.16

## Data Availability

The original contributions presented in this study are included in the article material. Further inquiries can be directed to the corresponding author.
